# Editorial: Sirtuins From Bench to Bedside: How Far Are We?

**DOI:** 10.3389/fphys.2022.907501

**Published:** 2022-04-27

**Authors:** Jose Thaiparambil

**Affiliations:** Houston Methodist Cancer Center, Houston, TX, United States

**Keywords:** inflammation, metabolism, cvd, neurodegeneration, genomic instability

Sirtuins (SIRT1-7) regulate diverse physiological processes including metabolism, oxidative stress, DNA repair, protein synthesis, inflammation, and cell death. Recent literature shows that dysregulation of sirtuins could lead to heart failure, neurological disorders, and cancer. However, the mechanisms of sirtuin regulation in diverse cellular processes and their clinical utility are not well understood; five reviews and a research report examining these topics are discussed here.

Watroba and Szukiewicz focused on the involvement of sirtuins in neurodegeneration, the inflammatory response, metabolic syndrome, DNA damage, and genomic instability as hallmarks of aging and cancer incidence. It explains that caloric restriction could play a role in anti-aging by activating sirtuins, which stimulate nicotinamide adenine dinucleotide (NAD+) biosynthesis, raising the level of intracellular NAD+. This is a hot topic due to public and scientific interest in caloric restriction to reduce body weight and slow aging and associated diseases. SIRT1 induction in neurons results in mTOR inhibition that promotes neurite outgrowth and increased degradation of the toxic protein aggregates like beta-amyloid that are responsible for neurodegeneration. As there are currently no drugs available to prevent neurodegeneration, sirtuin isoforms should be further explored for neuroprotection. Authors discussed some tissue-specific functions of sirtuins like anti-apoptotic, anti-inflammatory, and anti-fibrotic actions in the kidneys, liver, heart, skeletal muscle, hematopoietic system, and immune system.

The genetic and pharmacological modulation of sirtuins in cancer, neurological, and cardiovascular diseases (CVD) was the focus of Hong and Lin. The reviewers highlighted modulators strategically designed to directly bind sirtuins and generate beneficial effects in cellular and animal models of human disease. Sirtuins are involved in a plethora of biological pathways and could play both tumor suppressor and activator roles. SIRT1, SIRT2, SIRT3, and SIRT5 inhibitors were developed to prevent cell proliferation and tumor growth, and numerous pan-sirtuin inhibitors also decrease cancer cell proliferation. Many SIRT2 inhibitors ameliorate the symptoms from neurological disease. In addition, several sirtuins have been connected to CVDs, including vascular aging, atherosclerosis, and cardiac hypertrophy. Although the authors noted a few contradicting reports, some general trends can be extracted from the literature. SIRT2 and SIRT5 inhibitors show consistent and promising effects in treating cancers and SIRT2 inhibitors show beneficial effects in neurological diseases. Conversely, SIRT1 and SIRT6 inhibitors aggravate CVDs, showing the need for reliable SIRT1 and SIRT6 activators. These trends support the development of sirtuin modulators with enhanced potency and selectivity, to validate preclinical data and potentially treat various human diseases.

Wang et al. summarized the critical role of sirtuins in protecting against cardiovascular and metabolic diseases through functions including suppressing inflammation, improving lipid profiles, and scavenging oxidative stress. Another interesting observation is that, in addition to activating sirtuins, boosting their common co-substrate NAD + appears a straightforward and effective solution for pan-sirtuin activation. The authors also describe a report showing that caloric restriction is associated with increases in NAD + levels, sirtuin expression, and sirtuin activity in the target tissue (e.g., adipose tissue for obesity, aortae and heart for CVD). The similar state induced by caloric restriction as that induced by antidiabetic drugs may be mediated in part by sirtuin activation. This study establishes the great research potential of caloric restriction related to sirtuin activation.

Ministrini et al. linked SIRT1 to cellular senescence and aging through its role in endothelial dysfunction and consequent CVD. Sirtuins regulate multiple cell functions including mitochondrial respiration, redox balance, apoptosis, cell signaling, and inflammation. These properties contribute to the well-known effect of SIRT1 in preventing cell senescence and prolonging lifespan in animals. Since the cardiovascular system is severely affected by aging, and atherosclerotic CVD is the most common age-related disease in industrialized countries, the pathophysiological role of SIRT1 in CVD has been intensively investigated in the past 20 years. These studies clearly support a protective role of SIRT1 toward endothelial integrity and function. Accordingly, enhancing the activity of SIRT1 through pharmacologic and non-pharmacologic interventions is a promising approach in the prevention and treatment of endothelial dysfunction and atherosclerotic CVD, although convincing evidence in humans, particularly in CVD patients, is missing. Clinical and pre-clinical studies on this promising topic are ongoing.

In a brief research report, Greiten et al. showed that depletion of SIRT6 elicits endothelial dysfunction in young genetically altered mice and suggest that SIRT6 is a key regulator of vasomotor function in conduit vessels. They proposed that SIRT6 serves as a tonic suppressor of NAD(P)H oxidase expression and activation, as inhibition of NAD(P)H oxidase improved endothelial function in SIRT6 haploinsufficient mice. Overall, SIRT6 activation and/or histone acetyltransferase inhibition may be useful therapeutic approaches to reduce endothelial dysfunction and combat age-associated CVD. This is the first study reporting that global genetic SIRT6 reduction is a major determinant of histone acetylation and oxidative stress in the vasculature, and that SIRT6 deficiency results in significant endothelial dysfunction due to increases in NAD(P)H oxidase-derived reactive oxygen species. Finally, the authors suggest that activators of SIRT6 could represent a novel class of therapeutic compounds with broad clinical utility.

Alternatively, Curry et al. discussed small molecule sirtuin regulators that have advanced into clinical trials for future drug development, describing their discovery, mechanism of action, pharmacokinetics analysis, formulation, and outcomes observed in the trials. They explain the outcomes of SIRT1 activator resveratrol (RSV) and related activators in clinical trials for conditions like cancer, CVD, neurological disorders, diabetes, inflammatory diseases, and metabolic disorders. Another SIRT1 inhibitor, EX-527, is a potential therapeutic for Huntington’s disease, a neurodegenerative disease. Also discussed is quercetin, a flavonoid phytoestrogen and SIRT1 activator with activity in the management of brain, blood, salivary gland, and uterine cancers.

Targeting sirtuins by drugs or genetic modulation has great therapeutic potential. Further research on sirtuin-targeting activators and inhibitors will unravel the underlying mechanisms, and development of the lead compounds will bring sirtuin regulators into the clinic for treating diseases with considerable unmet medical needs.

